# Supplementary material for: Optimizing bio-orthogonal non-canonical amino acid tagging (BONCAT) for low-disruption labeling of Arabidopsis proteins in vivo
Source: Plant Physiol. 2025 Nov 7;201(1):kiaf573. doi: 10.1093/plphys/kiaf573 (PMC13221635; doi:10.1093/plphys/kiaf573)
Supplement: kiaf573_Supplementary_Data [file kiaf573_supplementary_data.zip › SUPPLEMENTARY FIGURES.pdf]

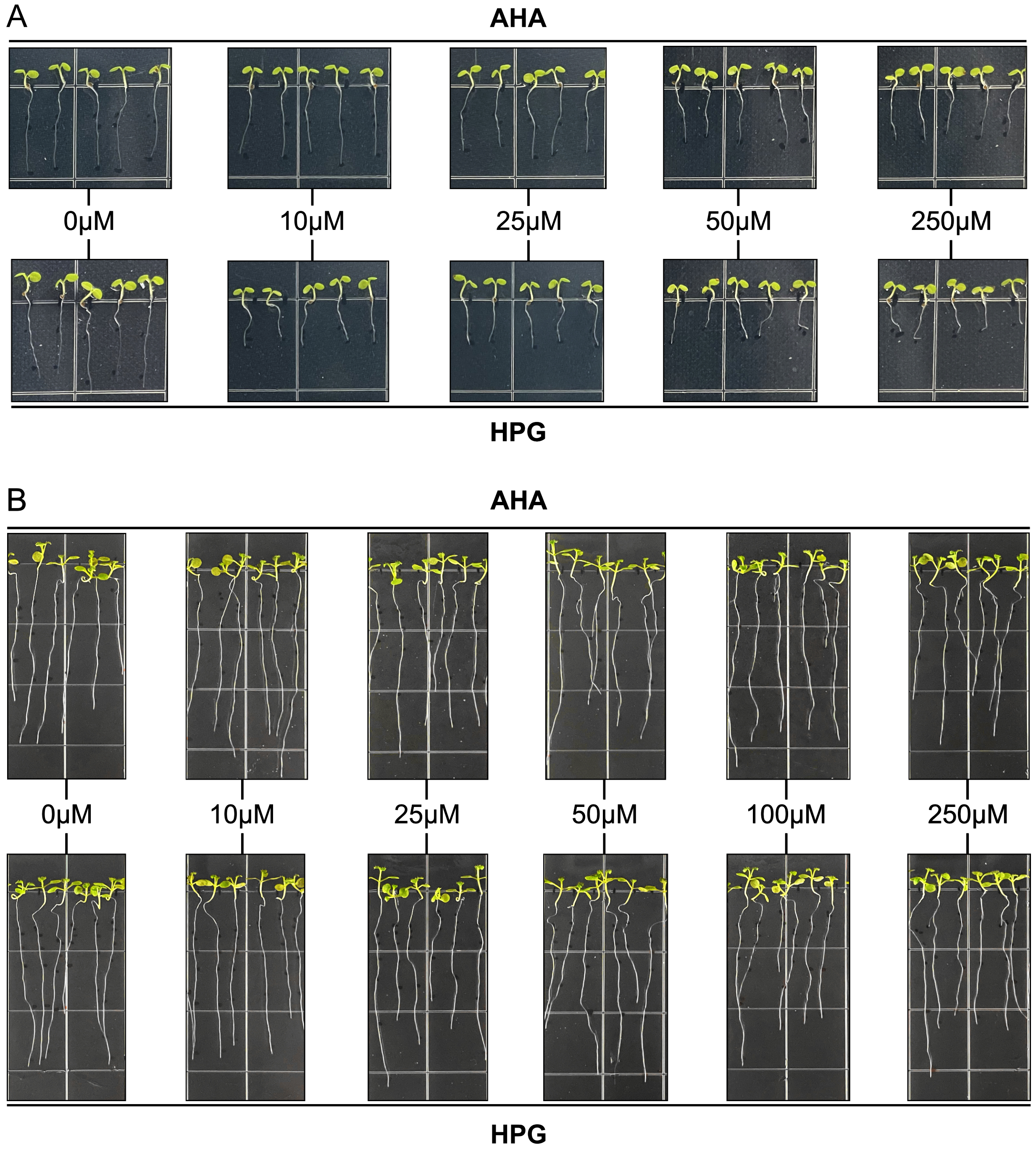

Supplementary Figure S1. Representative images of seedling samples during AHA and HPG viability assays. All seedlings were grown vertically on 0.5X MS agar for 5d before transplanting. Controls are ddH_2O. Images were taken at the end of each growth assay. A) Root length images at 3d post-transplant of seedlings with chronic exposure to AHA or HPG through 0.5X MS agar containing NCAAs. Full data is represented in Figure 3A. B) Root length images at 7d post-transplant of seedlings with brief (30min) exposure to AHA or HPG followed by wash and re-plating on 0.5X MS agar to verify incorporation phase viability. Full data is represented in Figure 3B and C.

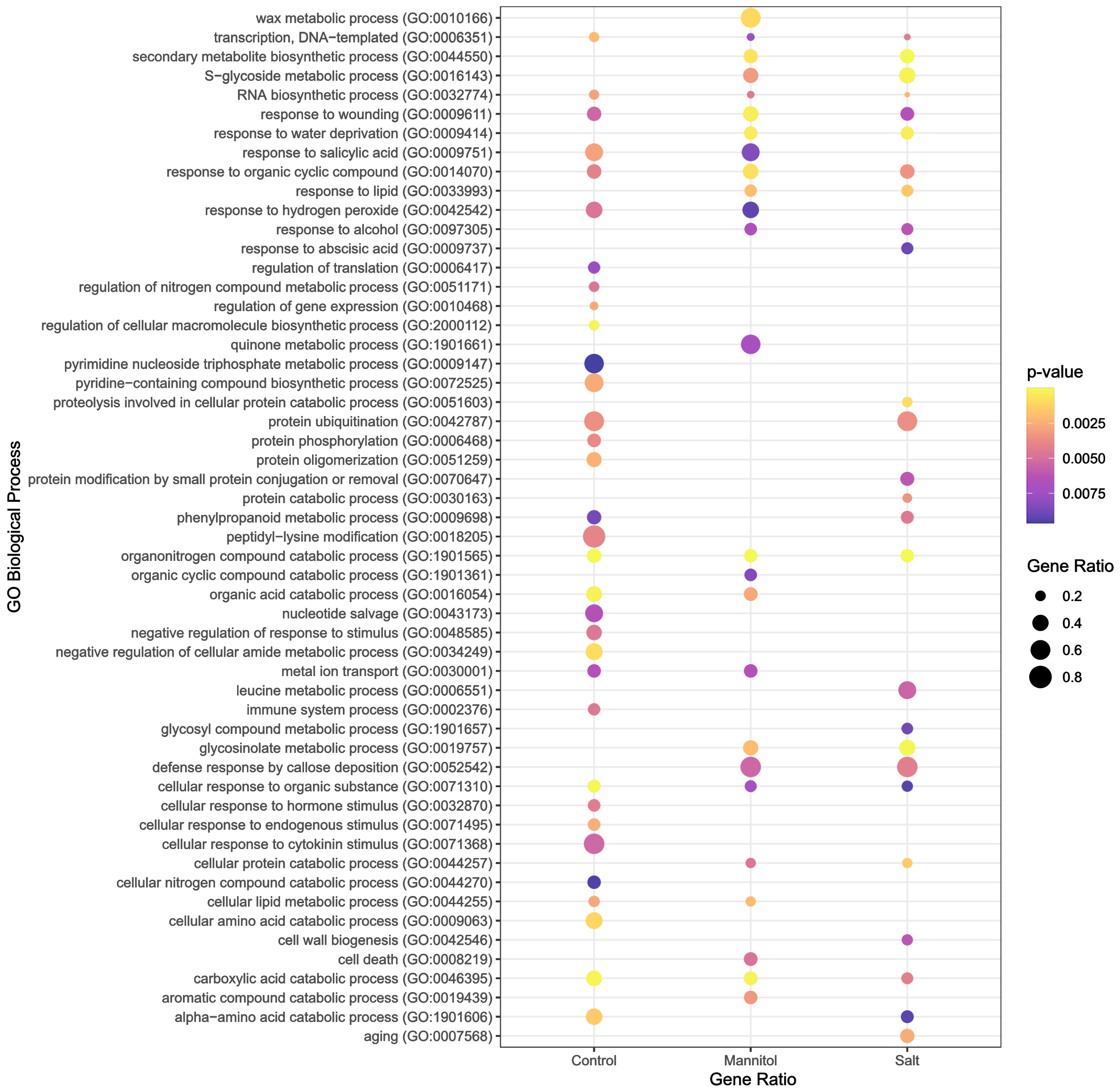

Supplementary Figure S2. Enlarged gene ontology of high stress set AHA-treated seedlings. GO terms with p-value < 0.01 (Benjamini-Hochberg parent-child union) for biological processes of proteins with Log_2 fold change > 0.58 over unlabelled controls in 50μM AHA-treated unstressed control, 150mM salt stressed, or 300mM mannitol stressed seedlings are represented.

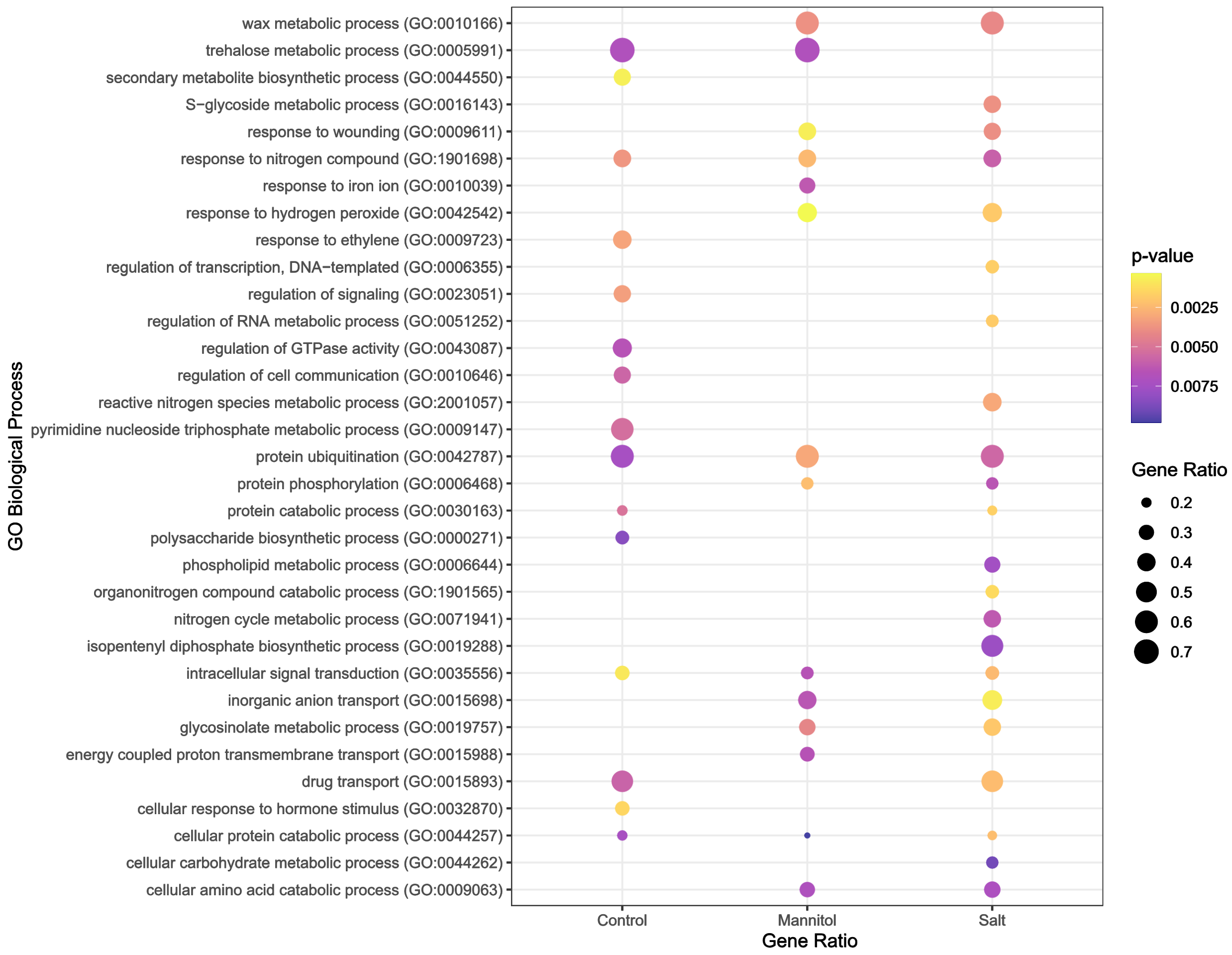

Supplementary Figure S3. Enlarged gene ontology of low stress set AHA-treated seedlings. GO terms with p-value < 0.01 (Benjamini-Hochberg parent-child union) for biological processes of proteins with Log_2 fold change > 0.58 over unlabelled controls in 50μM AHA-treated unstressed control, 50mM salt stressed, or 100mM mannitol stressed seedlings are represented.

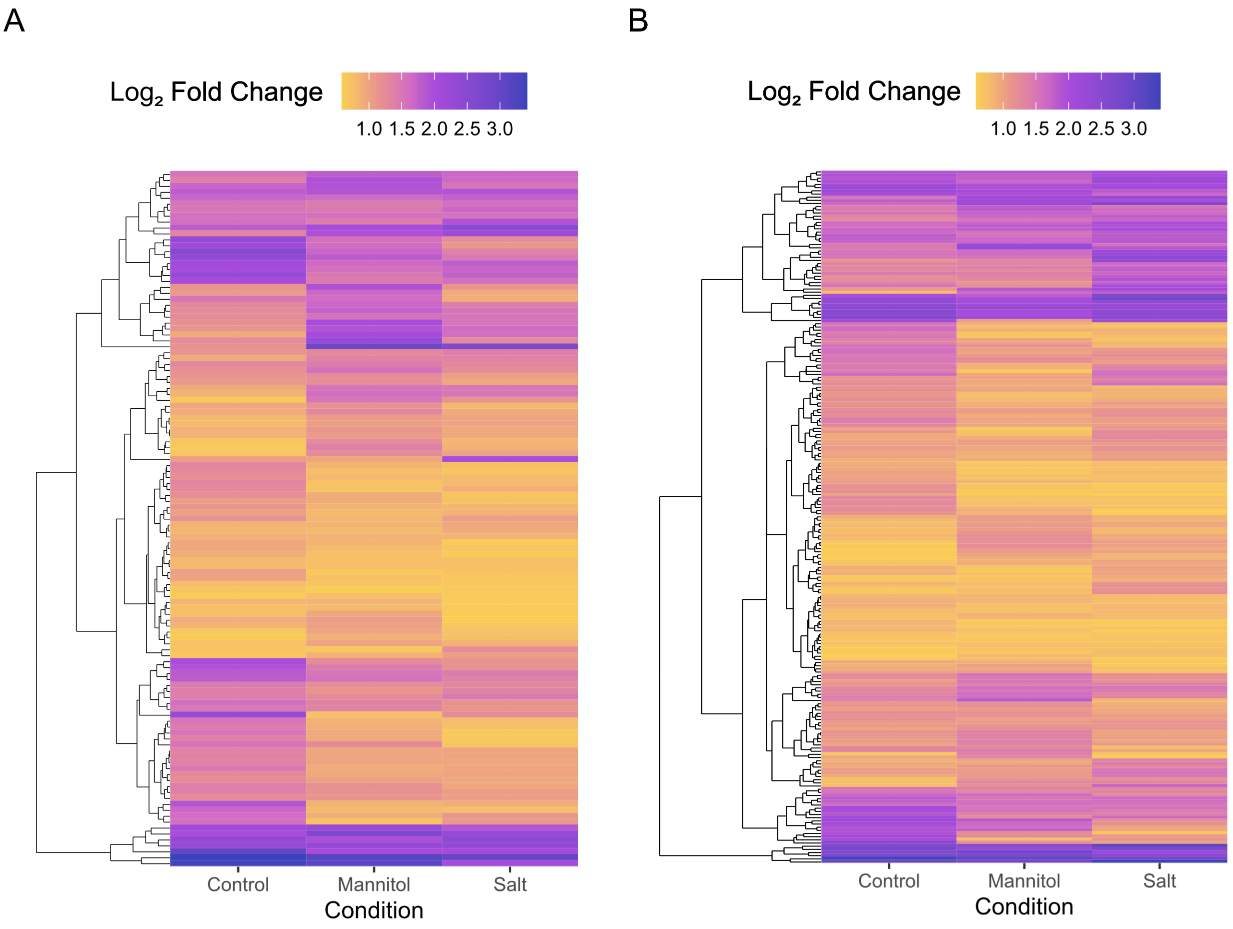

Supplementary Figure S4. Heatmap representation of shared proteins between salt and mannitol stressed AHA-treated seedlings. A) Euclidian distance-clustered heatmap of differentially expressed proteins that were significantly enriched (>0.58 Log$_2$ fold change) in all 3 AHA-treated conditions over untreated control in the high stress conditions, without Class I hits that were absent in the negative control (n = 117). B) Same as A, but for only terms enriched in all 3 groups in low stress conditions (n = 219).

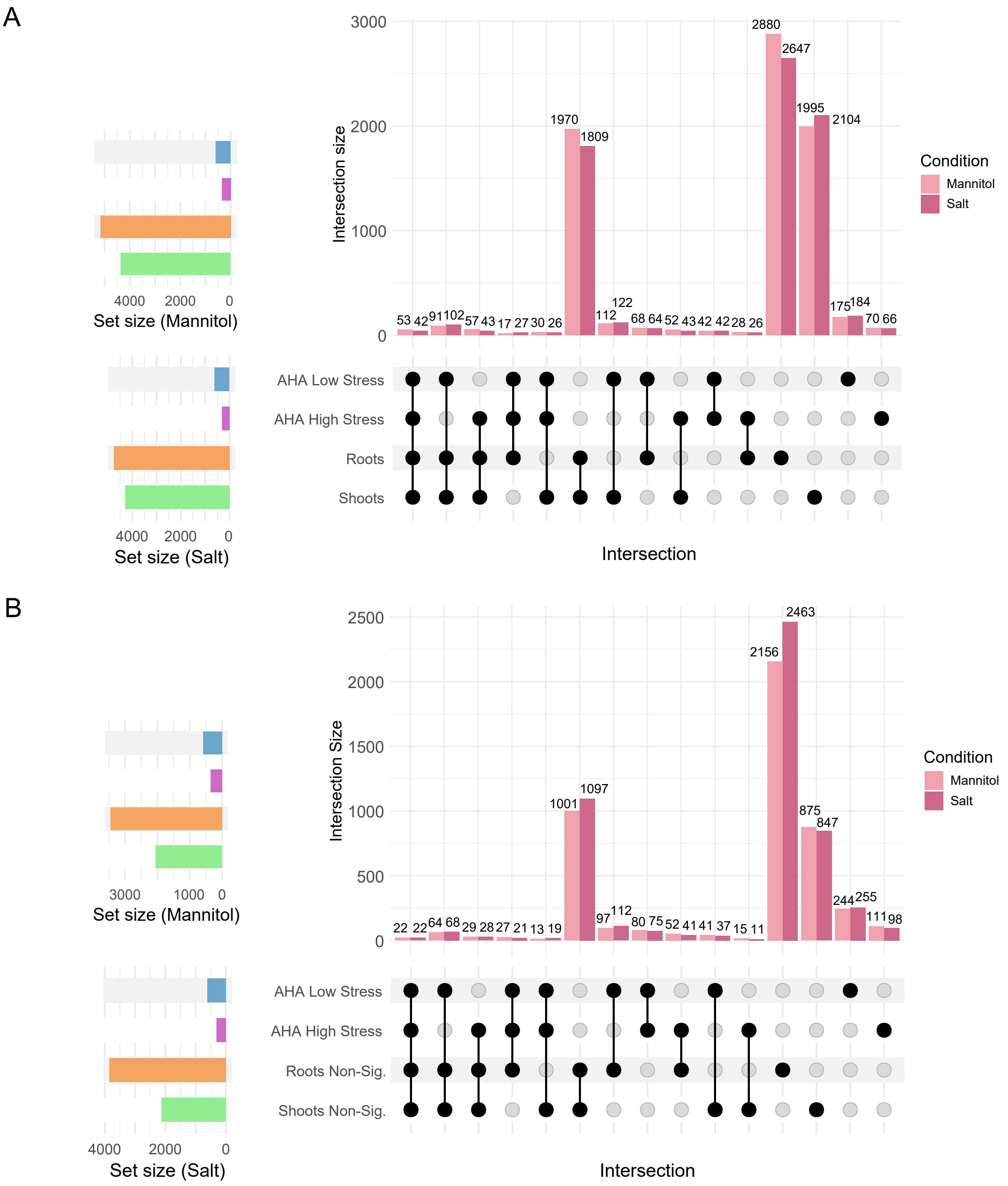

Supplementary Figure S5. Comparison of salt and mannitol stress data between previous whole proteome analysis and AHA-tagged enrichments. Data for whole proteome analysis was obtained by Rodriguez Gallo et al. (2023). **A)** Comparison of significantly changing root or shoot proteins under salt and mannitol stress (Rodriguez Gallo et al 2023; > 0.58 Log2FC and q-value < 0.05, n = 5) to significantly changing click-enriched proteins from AHA-treated seedlings during salt or mannitol stress (> 0.58 Log2FC and q-value < 0.05, n = 4). **B)** Upset plot of non-significant (q > 0.05) proteins from roots or shoots detected by Gallo et al. (n = 5) compared with significantly changing click-enriched proteins from AHA-treated seedlings during salt or mannitol stress (> 0.58 Log2FC and q-value < 0.05, n = 4).

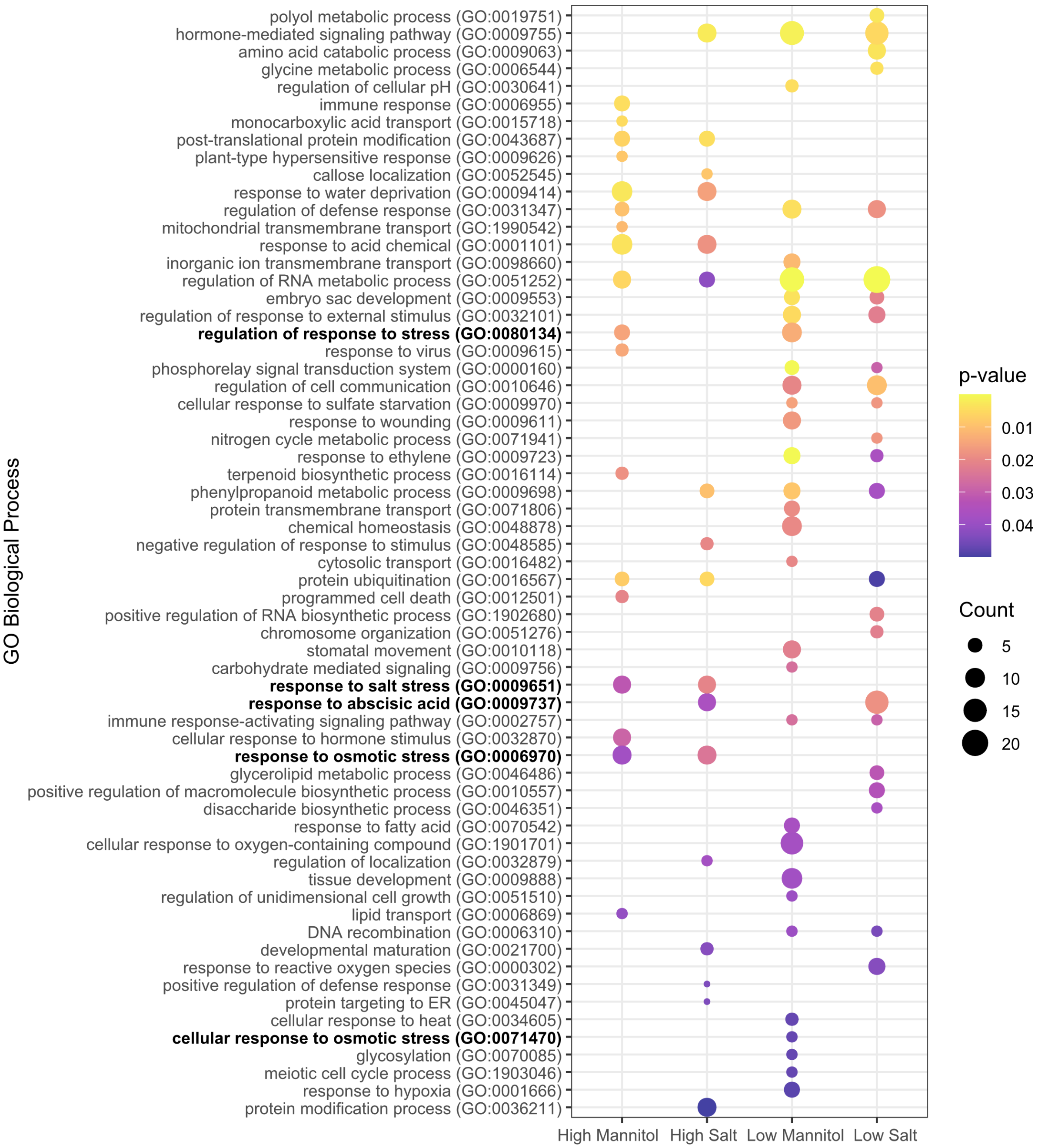

Supplementary Figure S6. Gene ontology of salt and mannitol stress data specific to AHA-tagged enrichments compared to previous whole proteome analysis. Gene ontology of biological processes (Benjamini-Hochberg parent-child union, $p < 0.05$) for 'high stress' (300mM mannitol and 150mM salt) and 'low stress' (100mM mannitol and 50mM salt) conditions. All proteins enriched in a stress condition over unlabelled controls in AHA enrichments but not previously detected as significantly changing proteins in the whole proteome analysis of roots or shoots under the same stressor by Rodriguez Gallo et al. (2023) were included as the foreground, with a background of all quantified proteins in the AHA enrichments. Bolded are terms directly related to stress response.
